# Mechanisms of Hsp90 regulation

**DOI:** 10.1042/BCJ20160005

**Published:** 2016-08-11

**Authors:** Chrisostomos Prodromou

**Affiliations:** * Genome Damage and Stability Centre, Science Park Road, Falmer, Brighton, East Sussex BN1 9RQ, U.K.

**Keywords:** chaperones, co-chaperones, heat-shock response, HSF1, Hsp90, post-translational modification

## Abstract

Heat shock protein 90 (Hsp90) is a molecular chaperone that is involved in the activation of disparate client proteins. This implicates Hsp90 in diverse biological processes that require a variety of co-ordinated regulatory mechanisms to control its activity. Perhaps the most important regulator is heat shock factor 1 (HSF1), which is primarily responsible for upregulating Hsp90 by binding heat shock elements (HSEs) within Hsp90 promoters. HSF1 is itself subject to a variety of regulatory processes and can directly respond to stress. HSF1 also interacts with a variety of transcriptional factors that help integrate biological signals, which in turn regulate Hsp90 appropriately. Because of the diverse clientele of Hsp90 a whole variety of co-chaperones also regulate its activity and some are directly responsible for delivery of client protein. Consequently, co-chaperones themselves, like Hsp90, are also subject to regulatory mechanisms such as post translational modification. This review, looks at the many different levels by which Hsp90 activity is ultimately regulated.

## INTRODUCTION

Hsp90 (heat-shock protein 90) accounts for 1–2% of the cellular protein and rises to 4–6% in stressed cells [1–4]. Levels of Hsp90 in cells are dependent on the master HSR (heat-shock response) regulator HSF1 (heat-shock factor 1), which is subject to a complex set of regulatory processes. Additionally, Hsp90 is regulated by other mechanisms that have an impact on its transcription, and is subject to post-translational modification and regulation by co-chaperones. Human cells contain a constitutively expressed Hsp90β (*HSP90AB1*) and a heat-inducible Hsp90α (*HSP90AA1*) [5], that were separated ∼500 million years ago, but still maintain 86% amino acid sequence identity [6]. Despite high conservation the proteins display different functions [7]. Interestingly, Hsp90α is not essential in mammals, whereas Hsp90β is, suggesting that Hsp90β is involved in processes that maintain viability, whereas Hsp90α is involved in more adaptive roles [8,9]. Hsp90 is responsible for the maturation of key signalling proteins including regulatory kinases [10–12], steroid hormone receptors [13] and transcription factors [14]. Hsp90 has been implicated in the assembly and disassembly of protein complexes [15] and can suppress phenotypic variation [16–19]. Collectively, Hsp90α and Hsp90β interact with approximately 10% of the eukaryotic proteome [20], representing ∼2000 proteins [21], of which, to date, ∼725 experimentally determined interactions have been confirmed by direct protein–protein interaction experiments. This implicates Hsp90 in diverse biological processes [3] that necessitate a wide range of mechanisms to regulate its function. The present review examines the major mechanisms, from HSF1 to co-chaperones, which regulate cytoplasmic Hsp90s.

## STRUCTURE OF Hsp90

**Figure 1:**
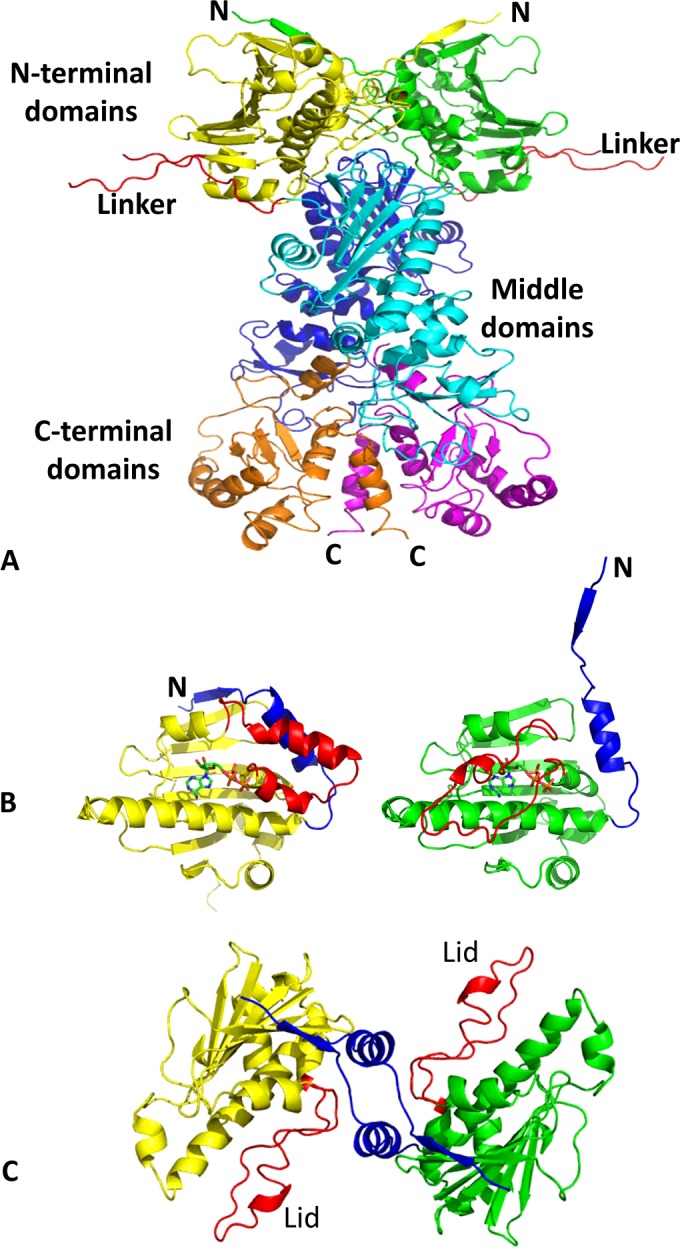
Structure and conformational change in Hsp90 (**A**) The Hsp90 dimer in a closed conformation involving transient dimerization of the N-terminal domains. N-terminal domains, yellow and green; middle domains, blue and cyan; C-terminal domains, orange and magenta; charged linker, red. (**B**) Conformation of the lid and N-terminal segment of the N-terminal domains of Hsp90 in the open undimerized state (left-hand panel, yellow) and the closed dimerized state (right-hand panel, green). Lids, red; N-terminal segment, blue. (**C**) The closed transient N-terminal dimerized state of Hsp90 (yellow and green). Lids, red; N-terminal segment, blue. N-terminal dimerization involves movement of the N-terminal segments of the N-terminal domains and association with the closed lid segments and with the neighbouring N-terminal domains.

**Figure 2:**
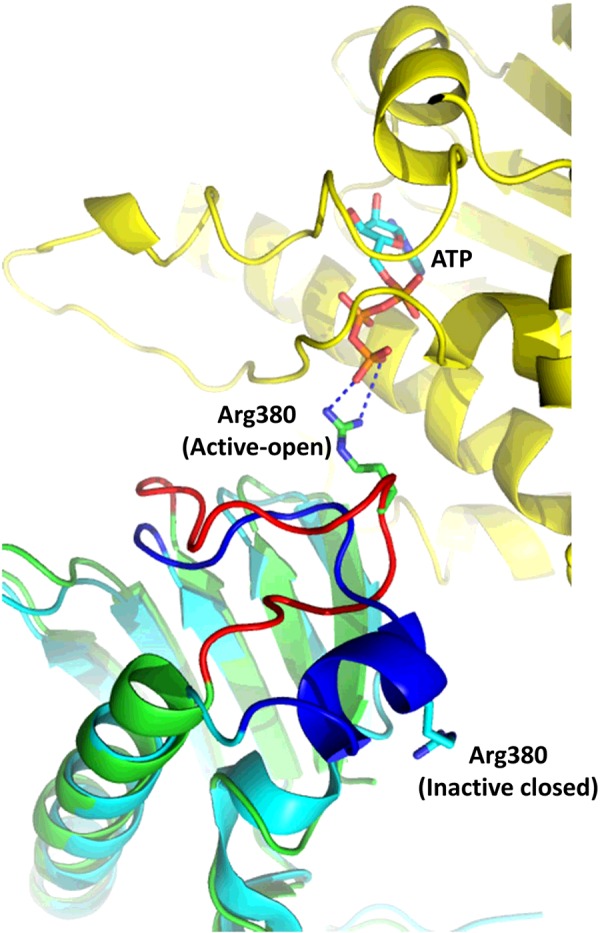
Conformation of the catalytic loop of Hsp90 The N-terminal domain of Hsp90 is shown in yellow. The middle domain is represented by two superimposed molecules of Hsp90 (cyan and green), one with a closed inactive catalytic loop (blue) and the other with an open active state (red) that interacts with the bound ATP, which is shown as a stick model. Arg^380^ of the catalytic loop is either interacting with the ATP (active state) or is held in an inactive state. Broken blue lines represent hydrogen bonds.

**Figure 3:**
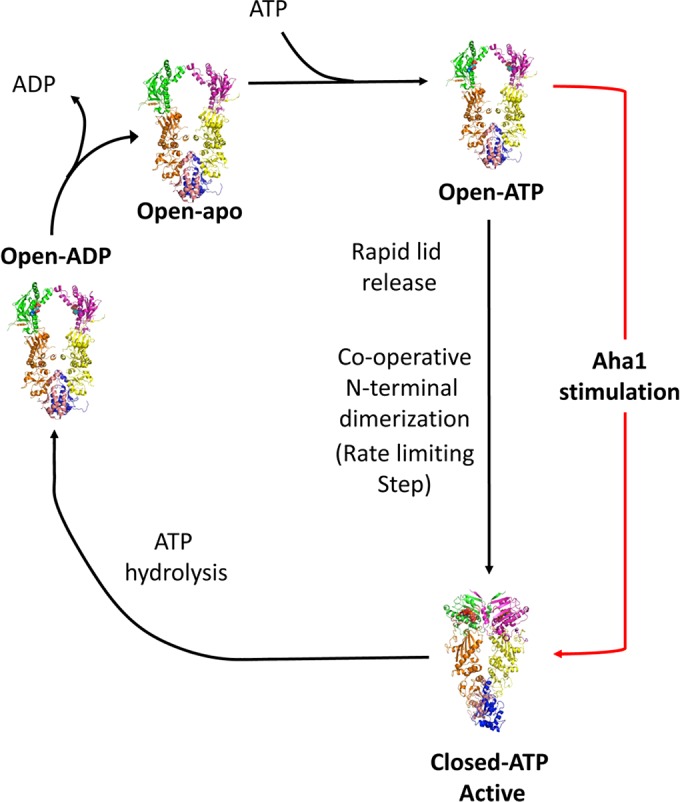
The Hsp90 chaperone cycle ATP binding triggers transient N-terminal dimerization, through conformational changes in the N-terminal domain of Hsp90 including those of the lid and N-terminal segment, and association with the catalytic loop of the middle domain. These motions act co-operatively to form the catalytically active closed state of Hsp90. Aha1, can accelerate the formation of this closed state by modulating the catalytic loop to an active open state. Once ATP is hydrolysed the N-terminal domains separate, the open inactive state of Hsp90 is formed and ADP is released. Hsp90 is now ready to enter the next cycle.

The structure and chaperone cycle of Hsp90 has been extensively reviewed elsewhere [22,23] and, consequently, only a basic description is provided in the present review. Hsp90 consists of three domains: an N-terminal dimerization domain, responsible for binding ATP, which is connected to a middle domain via an unstructured charged linker, and the C-terminal domain, which is responsible for the inherent dimerization of the protein, while the N-terminal domains undergo transient dimerization by binding ATP [24] (Figure 1). Binding of ATP promotes the movement of a lid segment within each N-terminal domain that locates over the bound ATP [25]. The movement of the lids exposes surface residues that are subsequently involved in transient dimerization of the N-terminal domains of Hsp90 (Figures 1B and 1C). ATPase activity of Hsp90 is achieved when the middle domain catalytic loop of Hsp90 moves to an open active state [26] (Figure 2). This loop possesses a conserved arginine residue (Arg^380^ in yeast), which interacts with the γ-phosphate of ATP, and thus promotes ATP hydrolysis by Hsp90. The active conformation of the catalytic loop is modulated by the binding of the co-chaperone Aha1, which consequently stimulates the ATPase activity of Hsp90 [27]. The conformational changes, including lid closure and modulation of the catalytic loop, represent the rate-limiting step of the chaperone cycle of Hsp90 (Figure 3). Currently, the molecular detail by which the Hsp90 chaperone cycle brings about the activation and maturation of client proteins remains elusive.

## ACTIVATION OF HSF1 AND THE HEAT-SHOCK RESPONSE

HSF proteins are responsible for regulating the HSR [28–31], which is induced by a variety of stimuli including elevated temperature, bacterial or viral infection and oxidative stress [32]. As a master regulator of the HSR and a client protein of Hsp90, understanding HSF1 function is central to understanding the regulation of Hsp90.

**Figure 4:**
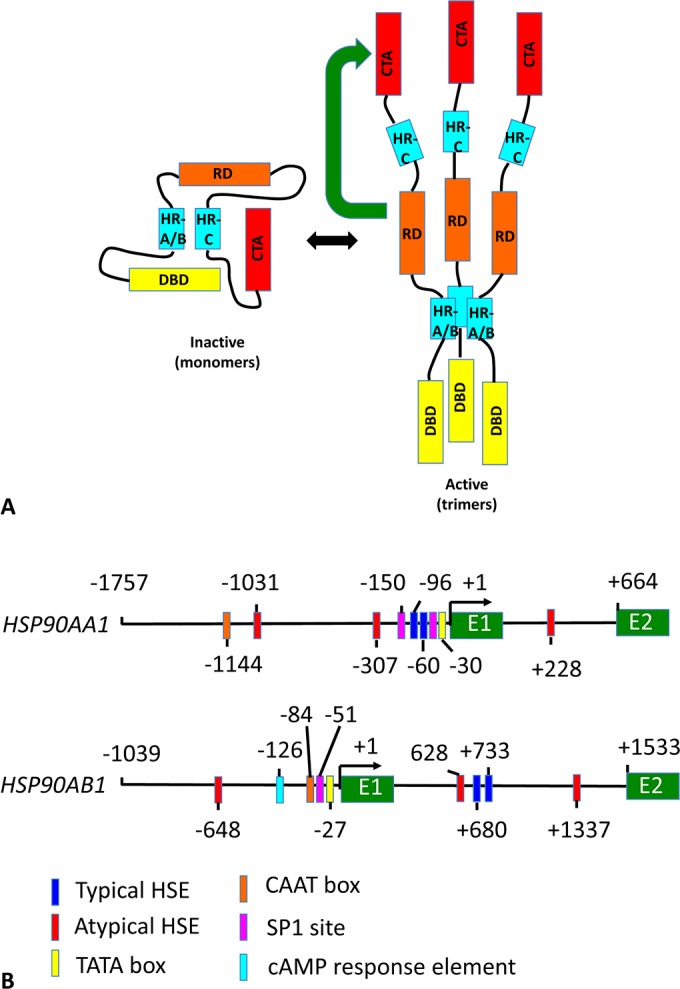
Domain structure of mammalian HSF1 and the promoter and upstream control elements of Hsp90-encoding genes (**A**) The domain structure of HSF1. The N-terminal DBD consists of the first 110 amino acids and is followed by the HR-A/B trimerization domain consisting of amino acids 130–203. The RD is encompassed by residues 221–383 and is followed by the HR-C region, residues 384–409, which negatively regulate the HR-A/B trimerization domain. The CTA domain consists of residues 410–529 and is negatively regulated by the RD (green arrow). Activation of HSF1 involves trimerization through the HR-A/B domains. Yeast HSF1 differs in that it also possesses an N-terminal transactivation domain that negatively regulates the RD. (**B**) Promoter and regulatory regions of human Hsp90-encoding genes. Sequences start from 5′ upstream regions, −1757 for *HSP90AA1* and −1039 for *HSP90AB1*, and end at the second exon. The approximate locations of various control elements are indicated and can be identified from the key. E1 and E2 are exons 1 and 2. The UPE (−125 to −37 bp) region of *HSP90AA1* confers a 10-fold up-regulation of the core promoter. The core promoter (−36 to +37 bp) of *HSP90AB1* confers constitutive expression, and the two typical HSE are responsible for maintaining a high level of constitutive expression.

All HSF proteins consist of a DBD (DNA-binding domain), a trimerization domain consisting of three leucine zipper repeats (HR-A/B), an HR-C region, which negatively regulates the HR-A/B domain, and a CTA (C-terminal transactivation) domain, which is negatively controlled by the central RD (regulatory domain) (reviewed in [30,33–35]) (Figure 4A). Yeast also possesses a novel NTA (N-terminal transactivation) domain [36]. The DBD of HSFs bind HSEs (heat-shock elements) that consist of a variable number of n**GAA**n units (reviewed in [37]) and the precise arrangement of these units can promote co-operativity between binding HSF trimers. The typical types of HSE approximate to three or more contiguous 5 bp repeat motifs (i.e. n**TTC**nn**GAA**nn**TTC**n).

The trimerization domain of HSF consists of two heptad repeats (HR-A and HR-B) that form a triple-stranded α-helical coiled coil [38]. In both *Drosophila* and mammalian HSF1 a third heptad repeat (HR-C) is responsible for intramolecular interactions with HR-A and HR-B, which maintain HSF1 in a monomeric state (Figure 4A), but is readily reversed by stress [39–41]. Trimerization is also stimulated by DBD intermolecular aromatic interactions between a tryptophan and a phenylalanine residue [42], and in mammals by two cysteine residues that form a disulfide bond in response to stress [42,43].

The CTA domain is predominantly unfolded, but its α-helical content increases due to elevated temperature resulting in the hyperphosphorylation of the protein. This is required for sustained increases in transcriptional activation when a single HSF trimer is bound to three n**GGA**n units [44,45], but dispensable when HSF1 trimers bind co-operatively to HSEs consisting of four or more n**GAA**n units [46–48], where each HSF1 molecule binds at least two of the four n**GAA**n units. In yeast, the NTA domain is unstructured [49] and appears to mediate transient activation of HSF1, suggesting that it behaves as a negative regulator of the CTA domain and that this domain is not wholly sufficient for stress-mediated HSF1 activation [50,51]. The CTA domain drives increasing levels of sustained promoter activity over normal growth temperatures (15–33°C), but transient activity, directed by the NTA domain, is induced over a higher and narrower temperature range (34.5–39°C) [52].

During stress, denatured protein levels accumulate [28], which triggers the conversion of the cytoplasmic non-DNA-binding HSF1 into a homotrimer that gains DNA-binding activity. Thus HSF1 is released from its repressed association with Hsp90 [53–55], undergoes homotrimerization [41] and translocation to the nucleus, where it binds HSE [56–58]. However, as yet it is incapable of enhancing transcription and has minimal transactivation competence [59–64]. The next phase involves a series of phosphorylations that transforms the HSF1 trimer into an active transcription factor (reviewed in [34,35]). This leads to a rapid up-regulation of Hsp90 as well as other chaperones and co-chaperones, including Hsp70, Hsp40 and Hsp27 [65].

Under normal conditions, many sites in the RD of HSF1, including Ser^230^, Ser^303^, Ser^307^ and Ser^363^ are phosphorylated. Phosphorylations at Ser^303^, Ser^307^ and Ser^363^ are actually repressive to transcriptional activity, but, significantly, these can be overridden by stress [66,67] and appear to represent a stress-sensitive repressive system [34]. In contrast, phosphorylation at Ser^230^ appears to promote transcriptional activity of HSF1 and the basal phosphorylation state at Ser^230^ increases upon heat shock. However, the S230A mutation does not wholly repress heat-shock-induced transcriptional activity [68]. Another, very important stimulatory phosphorylation occurs at Ser^326^, which promotes the association of the co-activator Daxx, which appears to be an important mediator of HSF1 activation [69]. However, there are numerous kinases that are responsible for HSF1 phosphorylation events (reviewed in [34,35]), and these probably help to integrate signals from different signalling pathways. Although such phosphorylation events appear to be critical for HSF1 activation, recent evidence suggests that it is possible to uncouple the stress-inducible phosphorylation of HSF1 from its activation, suggesting that the phosphorylation signature of HSF1 alone is not an appropriate marker for HSF1 activity [70]. HSF1 has also been reported to be regulated by SUMOylation [71–74] and acetylation [75–77].

In addition to post-translational modifications, a number of other mechanisms exist that regulate HSF1. The RD of HSF1 carries an intrinsic ability to sense heat stress [78] and Hsp90 might play a role in repressing trimeric HSF1 [53,79]. Furthermore, Hsp70 and Hsp40 appear to be able to inhibit HSF1 transactivating activity, which might occur through the recruitment of a Hsp70-interacting transcriptional co-repressor, CoREST (co-repressor for element-1-silencing transcription factor) [80,81]. A ribonucleoprotein complex consisting of the translation elongation factor eEF1A (eukaryotic elongation factor 1A) and a constitutively expressed non-coding heat-shock RNA-1 RNA has also been reported to act as a HSF1 activator, and *in vitro* can promote the trimerization of HSF1 [82,83]. HSF1 can also associate with the molecular chaperone TriC [84], but whether Hsp90 is chaperoning the assembly of TriC or whether TriC plays a regulatory role in Hsp90 expression remains unknown.

In summary, HSF1 is the master regulator of Hsp90 levels in cells. Consequently, a complex series of regulatory mechanisms have evolved, including transcriptional, HSF1 trimerization, co-operative binding to HSEs, post-translational modification and the ability of HSF1 to detect stress directly. Together these mechanisms integrate a variety of signals that bring about appropriate changes in the level of Hsp90, as well as other heat-shock proteins, in cells.

## REGULATION OF Hsp90 GENE EXPRESSION

Once HSF1 is activated it up-regulates the HSR by binding to HSEs upstream of heat-shock genes, such as those encoding Hsp90, Hsp70, Hsp40 and small HSPs. In humans, the complement strand of chromosome 14q32.33 encodes the *HSP90AA1* gene (Hsp90α), while *HSP90AB1* (Hsp90β) is located at 6p21. Unusually for molecular chaperones, the human Hsp90-coding genes contain intron sequences and the translational initiation of both genes is located within the beginning of the second exon [85–87]. Within the first intron of *HSP90AB1*, there are two typical HSEs that are responsible for maintaining a high level of constitutive expression [88], in addition to two atypical HSEs (Figure 4B). A third atypical HSE site is located upstream of the transcriptional start site (Figure 4B). In contrast, *HSP90AA1* possesses two typical HSEs immediately upstream of the TATA box, two additional atypical sites further upstream and another atypical site within the first intron [89] (Figure 4B).

The core promoter (−36 to +37 bp) of *HSP90AB1* confers constitutive expression [89]. The promoter of *HSP90AB1* contains a CAAT box, an SP1 site, a TATA box (−27 bp) and a transcriptional start. In contrast, the promoter of *HSP90AA1* contains an SP1 (specificity protein 1) site, a TATA box (−30 bp) and the transcriptional start. The core promoter appears not to contain a CAAT box, although one has been located at −1144 bp (Figure 4B).

The UPE (upstream promoter element) (−125 to −37 bp) region of the *HSP90AA1* gene confers a 10-fold up-regulation of the core promoter. In contrast, the region −1377 to −848 bp has a negative effect on expression, but a further section, −1756 to −1377 bp, provides positive regulation that overcomes this negative effect. The UPE of *HSP90AA1* (−125 to −37 bp) contains an HSE at −96 to −60 bp, in which there is an array of 5 bp HSE motifs (g**GA**ggg**TTCTTC**c**GGA**ag**TTC**aa**GA**ggc**TTC**tg**GAA**a). The HSEs derived approximate to g**GA**ggg**TTC**t, c**TTC**cg**GAA** and g**TTC**aa**GA**ggc**TTC**tg**GAA**a, and this has been named the proximal HSE complex and meets the criterion of a typical HSE of at least three adjacent 5 bp motifs [89]. Within the upstream region there are a further five motifs, two located at −1031 to −1022 bp (c**GAA**aa**TTC**c) and another that matches the criterion of a typical HSE at −307 to −288 bp (g**GGA**cc**TTC**cc**GA**ga). Another HSE is found within the first intron at +238 to +247 bp (c**TTC**ag**GAA**t). During heat shock, induction of *HSP90AA1* is dependent on the coexistence of the distal HSE at −1031 to −1022 bp and the proximal HSE complex [89].

In comparison, the *HSP90AB1* gene possesses a CRE (cAMP-response element) (−126 bp) and a UPE that contains a CAAT box (−87 to −84 bp), an SP1 site (−51 bp), the TATA box at −27 bp and finally the transcriptional start (+1 bp) (Figure 4B). The *HSP90AB1* gene possesses one upstream atypical HSE at −684 to −634 bp (g**GAA**ac**T**g**C**tg**GAA**a) and four HSEs in the first intron of the gene. Two of these are typical HSEs (g**TTC**tg**GAA**ga**TTC**a at +680 to +695 bp and g**TTC**tg**GAA**gcttct at +733 to +747 bp), whereas the other two are atypical (c**TTC**ca**GA**tct**TTC**t at +628 to 642 bp and t**GAA**tt**TTC**a at +1337 to +1346 bp). The upstream HSE appears not to respond to heat shock, whereas the HSEs within the first intron play a vital role [88]. The intronic HSEs of *HSP90AB1*, relative to the atypical sites, are bound tightly by HSF1, and appear to be the most important for maintaining its high constitutive and heat-shock expression levels. Novel initiation sites within the first intron have also been identified for both genes [88,89].

**Figure 5:**
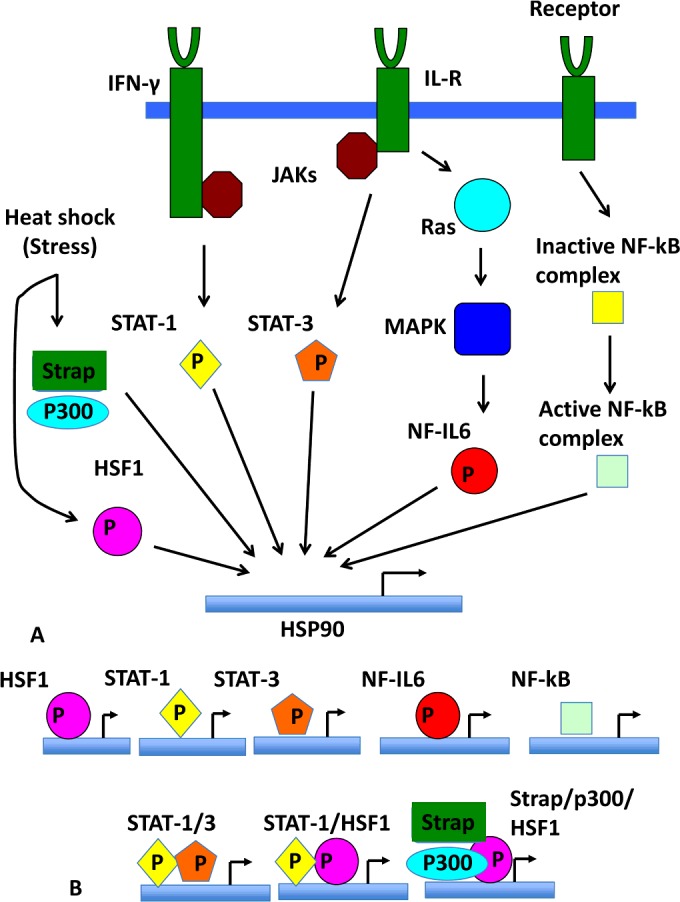
Integration of signalling pathways in the control of Hsp90 expression (**A**) Known signalling pathways that affect Hsp90 expression. The co-activator Daxx is not shown in the scheme, but is known to promote the activation of HSF1 [69]. (**B**) Possible binding scenario for transcriptional activators and cofactors that regulate *Hsp90* transcription. IFN-γ, interferon-γ; IL-R, interleukin receptor; JAK, Janus kinase; MAPK, mitogen-activated protein kinase.

In addition to the activation by HSF1, Hsp90β is up-regulated by the IL (interleukin)-6 transcription factors NF-IL6 (nuclear factor for IL-6) and STAT-3 (signal transducer and activator of transcription 3) [90]. Furthermore, IFN-γ (interferon-γ) activation of STAT-1 also up-regulates Hsp90β [90,91]. The binding sites for STAT-1 and STAT-3 appear to overlap with HSEs of HSF1 [90] (Figure 5), and significantly, the DNA-binding sites of STAT proteins (**TCC**N_2–4_**GAA**) are very similar to those of HSF1 (**TCC**nn**GAA**nn**TTC**) [37,92]. Additionally, STAT-1 and HSF1 can interact with each other and bring about strong transcriptional activation, whereas STAT-3 and HSF1 appear to be unable to interact and therefore antagonize each other, resulting in reduced expression of Hsp90β [90,91]. This leads to a rather complicated regulatory system where STAT-1 and STAT-3 activation leads to activation of *HSP90AB1* promoters, whereas interplay with HSF1 can modulate expression either up or down. It is likely that STAT-1 and STAT-3 play an important role in regulating Hsp90β under non-stressful conditions, and their interaction with HSF1 is a means by which they are able to integrate their responses with the stress response [90] (Figure 5). Furthermore, NF-IL6 has a similar, but not identical, DNA-binding consensus sequence (**TT**nn**G**n**AAT**) [93,94], but the significance of this, if any, is unknown.

The expression of human Hsp90 is augmented by Strap (stress-responsive activator of p300) [95], a transcription cofactor responsible for the control of the DNA damage response through a mechanism involving regulation of p53 activity [96,97] (Figure 5). Strap was reported as a heat-shock-inducible protein that forms a chromatin-associated complex with HSF1, and the co-activator p300, which has a histone acetylase activity and is required for activation by certain transcription factors [98]. It is thought that the ability to up-regulate Hsp90 expression might involve chromatin acetylation.

The *HSP90AA1* gene is under NF-κB (nuclear factor κB) regulatory control (Figure 5). The NF-κB family of transcription factors regulate the expression of a large variety of genes involved in a number of cellular processes such as inflammation, immune response, cell growth and development, and is activated as a response to a variety of signals, including cytokines, pathogens, injuries and other stressful conditions [99–106]. A single NF-κB putative consensus sequence (**GGTAGTTCCA**) was identified in the 5′-flanking region of the *HSP90AA1* promoter (but not in *HSP90AB1*) [5]. Binding of NF-κB to this site appears to up-regulate the expression of Hsp90α. Evidence suggests that HSP90α is required not only for the biosynthesis of the IKK (inhibitor of NF-κB kinase) [107], but also for the constitutive and inducible expression of IKK and NF-κB [107–111]. Thus NF-κB activity influences *HSP90AA1* gene expression, but reciprocal interactions between the activities of HSP90α and NF-κB are likely to constitute a regulatory loop that can influence cell survival and response to stressful agents.

In summary, the core promoter and the two typical HSEs in intron 1 are responsible for maintaining high constitutive expression of Hsp90β. The intronic HSEs also ensure that *HSP90AB1* is able to respond to heat shock. In contrast, the UPE, in which the proximal HSE complex is situated, confers a 10-fold up-regulation of the core promoter of *HSP90AA1*. The region at −1377 to −848 can promote a negative effect on expression that is overcome by another upstream region at −1756 to −1377. However, during heat-shock induction, the distal HSE at −1031 to −1022 bp together with the proximal HSE complex is responsible for the up-regulation of Hsp90α. Finally, a variety of other transcriptional regulators are used to integrate diverse cellular signals with the HSR.

## POST-TRANSLATIONAL REGULATION OF Hsp90

**Figure 6:**
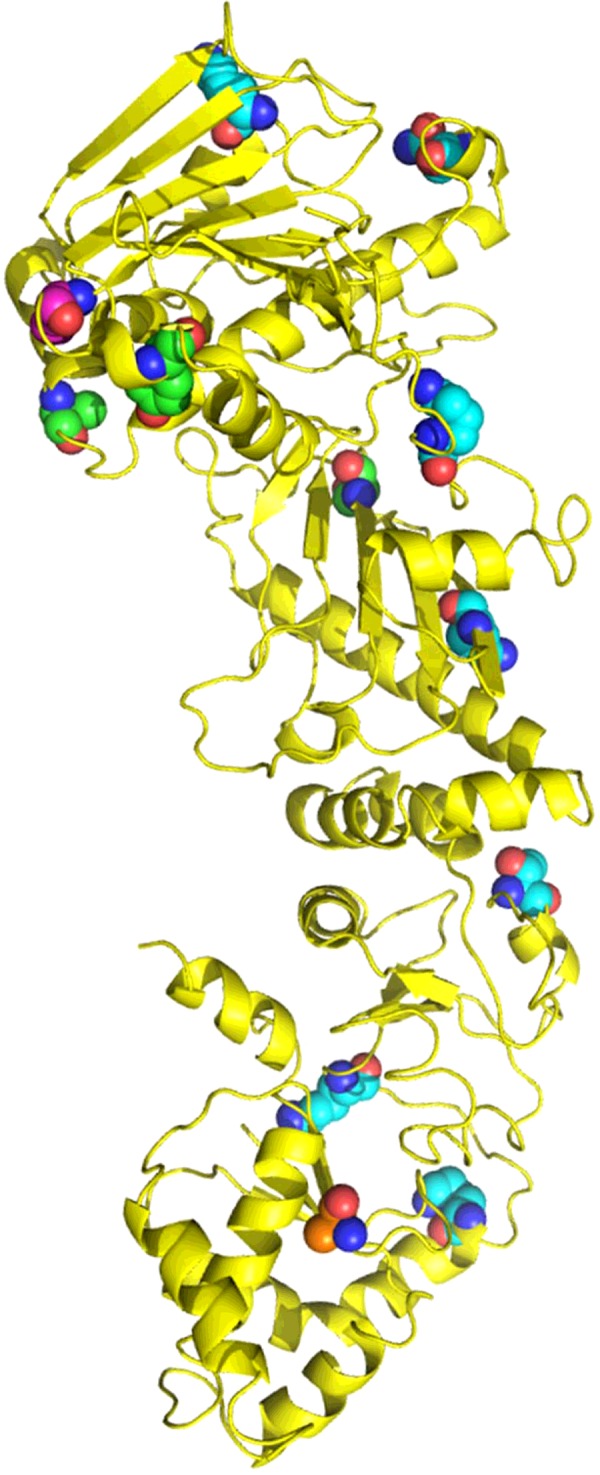
Post-translational modification of Hsp90 A single monomer of yeast Hsp90 (yellow) is shown in cartoon format. Amino acid residues from yeast and human Hsp90 that are post-translationally modified are shown as spheres and mapped to the correct location on yeast Hsp90. Modified amino acid residues for Hsp90α Ser^5^, Ser^7^ and Ser^234^ and Thr^725^ and Hsp90β Ser^255^ that are not represented in the yeast structure are omitted. Green spheres, amino acids that are phosphorylated; cyan spheres, amino acids that are acetylated, magenta spheres, amino acids that are SUMOylated; gold spheres, amino acids that are nitrosylated.

Because Hsp90 is involved in diverse cellular processes, it is perhaps not surprising that a vast array of post-translational modifications exist for both Hsp90α and Hsp90β that regulate their chaperone cycle. These include phosphorylation, acetylation, SUMOylation, methylation, ubiquitylation and S-nitrosylation and have been extensively reviewed in [7,112]. Post-translational modifications discussed in the present review are shown in Figure 6 in the context of the yeast protein and their effects summarized in Table 1. Although most of these modifications are common to both Hsp90α and Hsp90β, others, such as phosphorylation at Thr^5^ and Thr^7^ of Hsp90α in response to DNA damage [113,114], are specific. This offers a window into understanding not only the differential regulation of cytoplasmic Hsp90s, but also the different processes and functions that these proteins play within cells.

**Table 1:** Post-translational modifications occurring in yeast and human Hsp90 and their effects on the Hsp90 chaperone cycle and interactions with client proteins and co-chaperones yHsp90, yeast Hsp90.

Post-translational modification	Residue	Comment
Phosphorylation	Hsp90α Thr^5^ and Thr^7^	In response to DNA damage
	yHsp90 Thr^22^	Reduces interaction with Aha1 in yeast
	yHsp90 Thr^22^	Affects more than a single class of client protein maturation
	Hsp90α Thr^36^	
	Hsp90α Tyr^197^	Promotes the dissociation of Cdc37^p50^
	Hsp90α Ser^391^	May be required for ligand-independent epidermal growth factor receptor degradation
	Hsp90α Thr^725^	Determines the differential binding status of HOP and CHIP
	Hsp90α Ser^231^	Dissociation of AhR and destabilization of AhR
	Hsp90β Ser^226^ and Ser^255^	
Acetylation	Hsp90α Lys^69^, Lys^100^, Lys^292^, Lys^327^, Lys^478^, Lys^546^ and Lys^558^	Glutamine mutants show dcreased binding of nucleotides to Hsp90 (except K292Q)
	Hsp90α Lys^100^, Lys^292^, Lys^327^, Lys^478^, Lys^546^ and Lys^558^	Glutamine mutants show decreased binding with co-chaperones and, to a lesser extent, Hsp40
	Hsp90α Lys^69^, Lys^100^, Lys^327^, Lys^478^, Lys^546^ and Lys^558^	Glutamine mutants show reduced binding to CHIP, disrupted binding to Hsp70 and c-Raf (except K327Q)
	Hsp90α Lys^292^	Glutamine mutant shows decreased association with client proteins, including ErbB2, p60^v-src^, Raf-1, Hif1, mutant p53 and androgen receptor, and with some co-chaperones, including Aha1, CHIP and FKBP52
Nitrosylation	Hsp90α Cys^597^	Reported to inhibit Hsp90α ATPase activity
SUMOylation	yHsp90 Lys^178^, Hsp90α Lys^191^	Facilitates the association of Aha1 with Hsp90

Post-translational modifications have been seen to differentially regulate Hsp90 proteins in response to heat shock within different cellular environments [115,116]. Phosphorylation regulates not only Hsp90 activity directly, but also its ability to interact with chaperones, nucleotides and client proteins [113,115,117–120]. For example, phosphorylation of yeast Hsp90 at Thr^22^ (human Thr^31^) significantly reduces its interaction with the co-chaperone Aha1 [120,121]. Similarly, the phosphorylation of Hsp90α at Tyr^197^ by the Yes kinase promotes the dissociation of another co-chaperone, Cdc37^p50^, from Hsp90α [118]. Another phosphorylation that appears to be unique to Hsp90α occurs at Ser^391^, and may be required for ligand-independent epidermal growth factor receptor degradation probably through a PNCK (pregnancy-up-regulated non-ubiquitous calmodulin kinase)-dependent pathway [122,123]. The phosphorylation of Thr^725^ of Hsp90α determines the differential binding status of the HOP (Hsp70/Hsp90-organizing protein) and CHIP [C-terminus of the Hsc (heat-shock cognate) 70-interacting protein] co-chaperones. It appears that phosphorylation prevents the binding of CHIP, but enhances HOP interaction with Hsp90α [119]. In contrast, other phosphorylations within Hsp90α and Hsp90β appear to have effects only on one isoform. For example, the phosphorylation of Thr^90^ appears to signal the translocation of Hsp90α to the cell surface for secretion [124,125].

A major role played by phosphorylation of Hsp90 must be to differentially regulate Hsp90s activity with structurally diverse client proteins. For example, the phosphorylation of Ser^231^ (Hsp90α) or Ser^226^ and Ser^255^ (Hsp90β) result in the specific dissociation of AhR (aryl hydrocarbon receptor) and destabilization of AhR. In support of this, alanine mutations at these position up-regulated AhR and its association with Hsp90 [126]. In contrast with phosphorylations that differentially regulate Hsp90, others such as Thr^22^ in yeast Hsp90 and Thr^36^ in Hsp90α, appear to affect more than a single class of client protein as was evident using T36A and T36E Hsp90 mutants [120,121]. The kinases that carry out such phosphorylations have been reviewed in [112], and include double-stranded DNA protein kinase, B-raf, Akt, c-Src, protein kinase A, Swe^Wee1^ and casein kinase 2. Although much work has been carried out on determining the effects of Hsp90 phosphorylation, the phosphatases that act on Hsp90 to regulate phosphorylation are not well characterized, although yeast Ppt1 [PP5 (protein phosphatase 5) in humans], positively regulates Hsp90 activity through dephosphorylation [120,125,127].

Hsp90 is also subject to acetylation by p300, whereas deactylation occurs by a variety of HDACs (histone deacetylases) including HDAC1, HDAC6 and HDAC10 [128–132]. In one study seven acetylated lysine residues were identified in Hsp90α: Lys^69^, Lys^100^, Lys^292^, Lys^327^, Lys^478^, Lys^546^ and Lys^558^ [133]. Using glutamine as an acetylation mimetic, all of the mutants showed decreased binding for nucleotide, except K292Q, which displayed increased binding. The acetylation-mimetic mutants of Lys^100^, Lys^292^, Lys^327^, Lys^478^, Lys^546^ and Lys^558^ also displayed decreased binding with co-chaperones and, to a lesser extent, Hsp40. In contrast, the glutamine mutants at Lys^69^, Lys^100^, Lys^327^, Lys^478^, Lys^546^ and Lys^558^ showed reduced binding to CHIP, whereas all of the acetylation mutants, except K327Q, disrupted binding to Hsp70 and with c-Raf. The acetylated mimetic K292Q was also reported to show a decreased association with client proteins, including ErbB2, p60^v-src^, Raf-1, Hif1, mutant p53, and androgen receptor and with some co-chaperones, including Aha1, CHIP and FKBP52 (FK506-binding protein 52) [134]. Clearly, these results suggest that acetylation has a major impact on the regulation of human Hsp90.

Other modifications that have been reported include S-nitrosylation, ubiquitylation and SUMOylation. S-nitrosylation at Cys^597^ by nitric oxide (NO) was reported to inhibit Hsp90α ATPase activity and may represent a negative-feedback loop reducing the activation of eNOS (endothelial nitric oxide synthase), which is Hsp90-dependent [135]. The mechanism by which S-nitrosylation appears to affect Hsp90 might be through an allosteric mechanism bringing about the inhibition of its ATPase activity [136]. An increase in the ubiquitylation of human Hsp90 was shown to inhibit its function and cause a dissociation of client protein, including p53, Cdk4 (cyclin-dependent kinase 4) and Plk (Polo-like kinase 1), Akt1 and eNOS, which were subsequently degraded by the proteasome [137,138]. Swe1 phosphorylation of human Hsp90 signals its ubiquitylation and degradation by the proteasome, but the underlying detailed mechanism is unknown [139].

As with ubiquitylation of Hsp90, oxidative stress, which results in the direct oxidation of cysteine residues, also leads to client protein degradation, including Cdk4, cyclin D1, Raf-1, Akt and mutant p53 [140]. Finally, the asymmetric SUMOylation of the N-terminal domain of Hsp90 [Lys^178^ (yeast) and Lys^191^ (Hsp90α)] appears to facilitate the association of Aha1 with Hsp90 [141].

In summary, post-translational modifications of Hsp90 offer a means by which the chaperone cycle can be modulated. In particular, it aids the activation of a structurally diverse clientele by Hsp90 by allowing a means by which specific Hsp90s can be differentially regulated. Consequently, a vast array of post-translational modifications are involved in regulating Hsp90, but, for the most part, the regulatory mechanisms involved in these processes are still poorly understood.

## THE ATPase ACTIVITY OF Hsp90 AND REGULATION BY CO-CHAPERONES

Determining the exact mechanism by which Hsp90 hydrolyses ATP [24] is critical to understanding how client protein and co-chaperones regulate this activity. The crystal structure of the yeast N-terminal domain of Hsp90 in complex with AMP-PNP (adenosine 5′-[β,γ-imido]triphosphate), a non-hydrolysable analogue of ATP, provided the first direct evidence that Hsp90 was an ATPase [142–145]. Currently all Hsp90s are considered to be ATPases [146–149]. The structure of full-length yeast Hsp90 in complex with Sba1 provided the mechanistic detail by which the catalytically active state of Hsp90 forms, following the binding of ATP [22,25,150].

**Figure 7:**
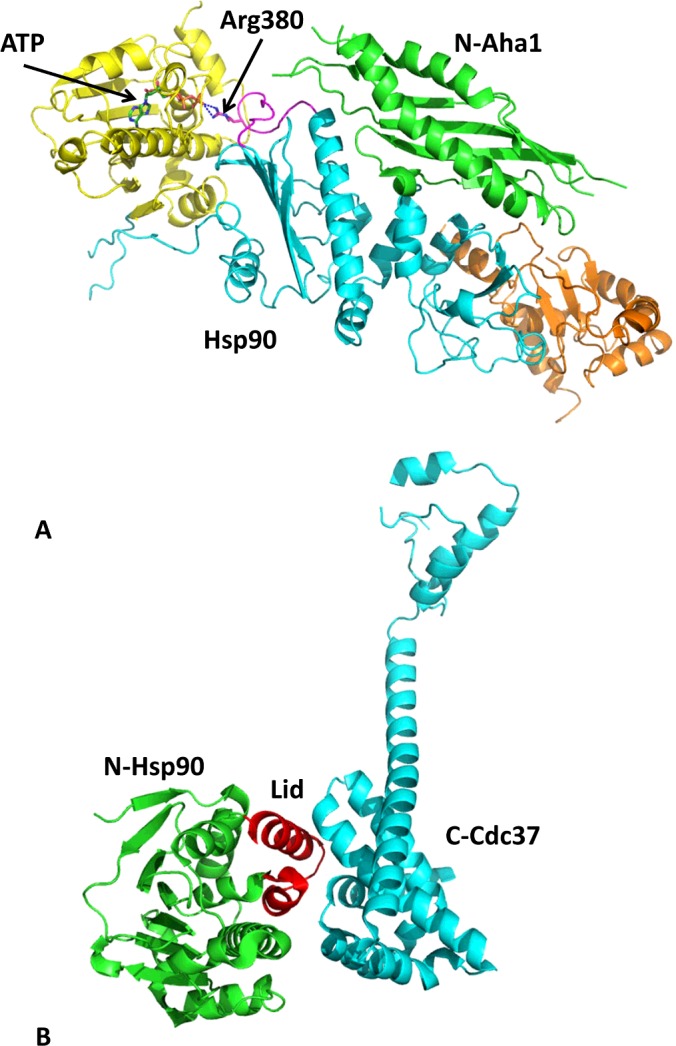
Structure of Hsp90–Aha1 and Hsp90–Cdc37^p50^ co-chaperone complexes (**A**) Structure of the Hsp90–Aha1 complex by superimposition of the middle domain of Hsp90 (cyan) in complex with Aha1 (green) on to the full-length structure of Hsp90 (N-terminus, yellow; C-terminus, gold). The binding of Aha1 causes the catalytic loop of Hsp90 (magenta) to move to its open state and allows Arg^380^ to interact with the γ-phosphate of ATP (green stick representation). Broken blue lines represent hydrogen bonds. (**B**) Structure of the N-terminal domain of Hsp90 (green) in complex with the C-terminal domain of Cdc37^p50^ (cyan). Cdc37^p50^ binds to the lid segment (red) of the N-terminal domains of Hsp90, preventing them from conformational movements that are required for the formation of the catalytically active state through N-terminal dimerization.

Rather than ATP hydrolysis [151], the rate-limiting step appears to be conformational change [24], which is now supported by recent kinetic analyses and structural data [25,152,153]. Previously, multi-exponential kinetics using FRET were interpreted as the formation of discrete conformational intermediates in the catalytic cycle [22,25,150,153–155]. However, recent work using yeast Hsp90 and 1-nm fluorescence probes based on photoinduced electron transfer, suggest a mechanism where closure of the lids, β-strand exchange and association of N- and M-domains share similar kinetics, and that these conformational changes act co-operatively to produce the catalytically active state ([156], and Andrea Schulze, Gerti Beliu, Dominic A Helmerich, Jonathan Schubert, Laurence H Pearl, Chrisostomos Prodromou and Hannes Neuweiler, unpublished work) (Figure 3). Thus binding of ATP rapidly releases the lid from its well-ordered open state to a dynamic intermediate. Full closure of the lid over the nucleotide-binding pocket occurs relatively slowly, but significantly in a co-operative manner with inter- and intra-subunit associations between the NTD and M-domains, and reciprocal exchange of the N-terminal β-strands. Furthermore, it appears that Aha1 remodels the catalytic loop in the M-domain of yeast Hsp90 into a conformation favouring engagement with ATP (Figure 7A), by stabilizing N/M-domain interactions [25,157], and by acting directly on the lid to accelerate closure. It was concluded that these conformational changes acting in concert limit the overall rate constant of ATP hydrolysis. With this in mind, we can now look at the effect of co-chaperones on the Hsp90 ATPase activity in a new light.

**Figure 8:**
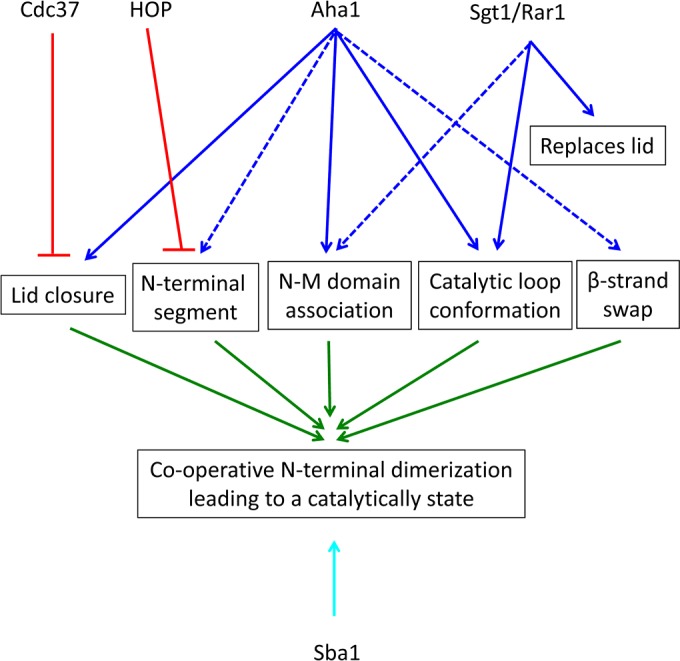
Co-chaperone pathways that modulate Hsp90 ATPase activity Cdc37^p50^ binds to the lids and prevents molecular rearrangement of Hsp90. HOP appears to interact with N-terminal segment of Hsp90 and thus may prevent N-terminal dimerization. Aha1 is able to interact with possibly all of the structural elements that lead to co-operative N-terminal dimerization of Hsp90. Sba1 interacts with the lid and N-terminal domains of Hsp90 and stabilizes Hsp90 in a closed state that displays a lower rate of ATP hydrolysis. Sgt1, together with Rar1, is unusual in that it activates Hsp90 in an open state and leads to a stable ADP-bound complex. Red and blue arrows indicate a mechanism resulting in the inhibition and activation of ATPase activity respectively. Broken blue arrows indicate interactions that might occur. The cyan arrow indicates a means by which the rate of ATPase activity is decreased. The green arrows indicate the co-operative nature of N-terminal dimerization.

The detailed biochemical and structural mechanistic effects of co-chaperones on the ATPase activity of Hsp90 has been reviewed in detail [22]. Consequently, I will only cover the mechanism of Hsp90 regulation in the light of the co-operative mechanism for N-terminal dimerization. One of the major roles played by co-chaperones, such as HOP/Sti1, Cdc37^p50^ and Sgt1, is the delivery of client protein to Hsp90 (see [22,23] for reviews). HOP, together with Hsp70, is responsible for delivering steroid hormone receptor to human Hsp90. In so doing, HOP, as well as the yeast orthologue Sti1, inhibits the ATPase activity of Hsp90 [158,159]. This probably represents a critical step that allows steroid hormone receptor to engage with human Hsp90. The primary binding site for HOP/Sti1 on Hsp90 is a highly conserved MEEVD motif that occurs at the extreme C-terminus of Hsp90 [160]. Additional contacts to the C-terminal, middle- and N-terminal domains of Hsp90 have been revealed by biochemical and structural studies with both the yeast and human protein [158,161,162]. Multiple interaction sites between HOP and human Hsp90 are supported by more recent evidence [163] and also suggests that monomeric HOP can bind to Hsp90 [163]. This supports the previous finding that Sti1 prevents N-terminal dimerization by interacting with the first 24 amino acid residues of yeast Hsp90, in addition to the conserved MEEVD motif of Hsp90 [162]. In the light of a mechanism involving co-operative N-terminal dimerization, the ability of Sti1/HOP to interact with the first 24 N-terminal amino acid residues could compromise this process (Figure 8). Such a mechanism is compatible with the observation that HOP, as a monomer, can inhibit Hsp90 ATPase activity, while simultaneously allowing access for the binding of immunophilins, which would promote progression of the chaperone cycle [159].

Cdc37^p50^ is involved in delivering client protein kinases to the Hsp90 complex, and, in a similar way to HOP and Sti1, it inhibits the ATPase activity of Hsp90 [164]. Thus Cdc37^p50^ binds between the N-terminal domains of Hsp90, interacting directly with the lids [165]. This prevents motions in the lids that would otherwise promote co-operative N-terminal dimerization (Figures 7B and 8). Exactly how Cdc37^p50^ exits the complex allowing progression of the chaperone cycle is currently unknown. However, the co-operative mechanism for N-terminal dimerization perhaps offers an explanation how this might occur (Andrea Schulze, Gerti Beliu, Dominic A Helmerich, Jonathan Schubert, Laurence H Pearl, Chrisostomos Prodromou and Hannes Neuweiler, unpublished work). The results of this study suggested that the co-chaperone Aha1 enhances the ATPase activity of Hsp90 by releasing the lid early in the catalytic cycle. It is therefore conceivable that Aha1 displaces Cdc37^p50^ by modulating the catalytic loop of the middle domain and by promoting the movement of the lids towards a closed state favouring N-terminal dimerization (Figure 8).

**Figure 9:**
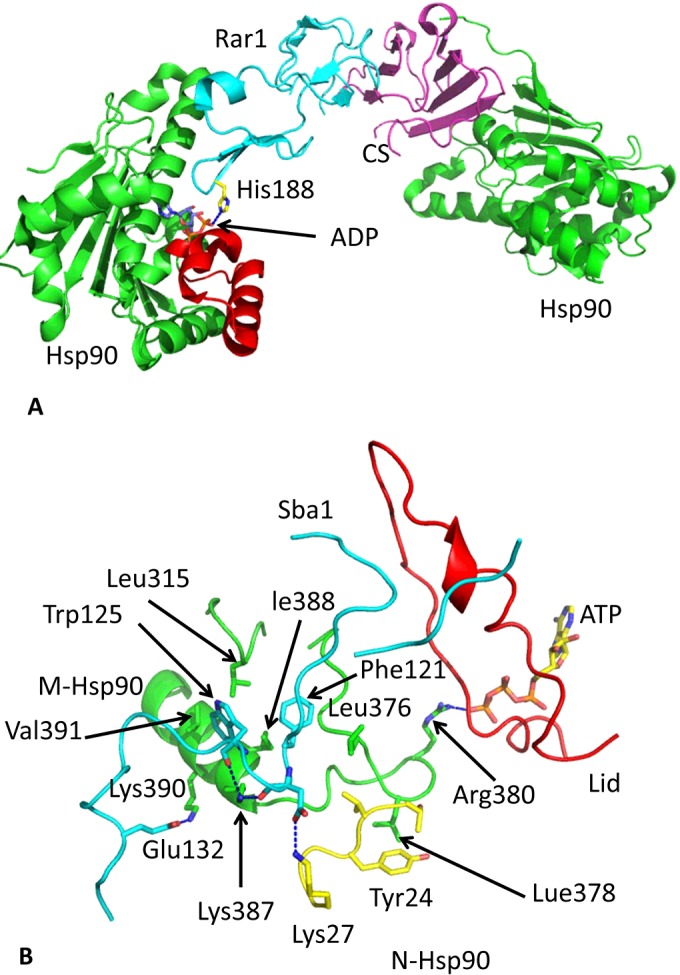
Structure of Hsp90–Rar1–Sgt1 and Hsp90–Rar1–Sba1 co-chaperone complexes (**A**) Structure of the N-terminal domains of Hsp90 (green) in complex with the CS domain of Sgt1 (magenta) and the CHORD II domain of Rar1 (cyan). Recruitment of Rar1 into the Hsp90 complex stimulates ATP hydrolysis producing a stable ADP-bound Hsp90 complex. The lid segment is shown in red and bound ADP in blue stick representation. Broken blue lines represent hydrogen bonds. (**B**) Structure of the closed conformation of Hsp90 in complex with Sba1 (cyan). Hsp90 is represented by the lid (red), the N-terminal segment of the N-terminus (yellow) and a segment of the middle domain (green). The bound ATP is shown as a yellow stick representation. Arg^380^ is seen to interact with the γ-phosphate of ATP in the catalytically active state of Hsp90. Broken blue lines represent hydrogen bonds.

Another co-chaperone that appears to be involved in client protein loading, by acting as hub for the formation of a variety of Hsp90 complexes, is Sgt1 [166–174]. Together with another co-chaperone, Rar1, Sgt1 plays a central role in the innate immunity response in plants. Sgt1 associates with the CBF3 kinetochore complex, with SCF E3 ubiquitin ligases, with plant R proteins and the related animal Nod-like receptors [175–182]. Sgt1 consists of three domains: an N-terminal TPR (tetratricopeptide repeat) domain, a middle CS (CHORD and Sgt1) domain, and a C-terminal SGS (Sgt1-specific) domain. The N-terminal TPR domain of Sgt1 is similar to other TPR domains that bind the conserved MEEVD motif of Hsp90. Surprisingly, however, the TPR domain of Sgt1 interacts directly with Skp1 [182], whereas the middle CS domain binds Hsp90 [182]. Structural details showing the CS domain interacting with the N-terminal domain of Hsp90 have been published [169]. Although the CS domain of Sgt1 is similar to that of Sba1/p23, unlike this latter co-chaperone, it does not regulate the ATPase activity of Hsp90 [169,182]. Instead, it recruits Rar1, a plant co-chaperone, or Chp1 and melusin in mammals, which, in the case of Rar1, weakly stimulates the Hsp90 ATPase activity [170]. The structure of the CS domain of Sgt1 and the CHORD II domain of Rar1 in complex with the N-terminal domain of Hsp90 has been published [170] (Figure 9A). In contrast with the activation of the ATPase activity by Aha1, the CHORD II domain of Rar1 stimulates the ATPase activity of Hsp90 in its open conformation. This promotes a stable ADP-bound open-state complex (Figure 9A). It appears that the CHORD II domain replaces the ATP lid and simultaneously modulates the middle domain catalytic loop to achieve activation of Hsp90. It is possible that the Rar1–Sgt1 complex might help promote N- to M-domain association of Hsp90, but currently this is unknown. The Hsp90–Sgt1–Rar1 complex perhaps mimics Hsp90’s catalytically active state, without N-terminal dimerization taking place. It therefore appears that Sgt1 and Rar1 stabilize the Hsp90 complex [178] by converting it into a long-lived ADP–Rar1–Hsp90–Sgt1 state [170].

Aha1 is the only co-chaperone known to strongly accelerate the ATPase cycle of Hsp90 [149,183]. Structural studies have shown that the N-terminal domain of Aha1 can modulate the middle domain catalytic loop of Hsp90, stabilizing it in an open active state (Figure 7A). Movement of the catalytic loop to its active state is now known to be required for co-operative dimerization by Hsp90 (Andrea Schulze, Gerti Beliu, Dominic A Helmerich, Jonathan Schubert, Laurence H Pearl, Chrisostomos Prodromou and Hannes Neuweiler, unpublished work), which would promote the ATPase activity of Hsp90. The observation that full-length Aha1 promotes the release of the Hsp90 lids early in the catalytic cycle is consistent with the co-operative nature of N-terminal dimerization. Furthermore, the binding of the C-terminal domain of Aha1 to the N-terminal domains of Hsp90 [184] is supportive of the idea that Aha1 promotes release of the lids early in the chaperone cycle (Andrea Schulze, Gerti Beliu, Dominic A Helmerich, Jonathan Schubert, Laurence H Pearl, Chrisostomos Prodromou and Hannes Neuweiler, unpublished work), and of the observation that full-length Aha1 is required for maximum stimulation of the ATPase activity of Hsp90 [149].

Sba1/p23 shows a higher affinity for the ATP-bound N-terminally dimerized state of Hsp90, rather than the apo or ADP-bound state [150,185]. Since Sba1 binds Hsp90 following the co-operatively driven mechanism of N-terminal dimerization, its role appears to be one of stabilizing the closed state of Hsp90. Sba1/p23, unlike co-chaperones that deliver client proteins to Hsp90 (Sti1, Cdc37^p50^ and Sgt1), therefore acts late in the Hsp90 chaperone cycle, which Sba1 appears to slow down, rather than totally inhibit [149]. In contrast, a more robust inhibition was reported for the human orthologue p23 [185]. The structure of the full-length yeast Hsp90 in complex with Sba1 and AMP-PNP explains the inhibitory effect of Sba1 binding [25]. The binding of Sba1 to the closed N-terminally dimerized domains of Hsp90 locks the N-terminal domains together, while simultaneously stabilizing the middle domain catalytic loop in an active conformation through a direct interaction (Figure 9B). Thus Sba1 temporally stabilizes the closed N-terminally dimerized state of Hsp90 by slowing its ATPase cycle [149].

In conclusion, the structural variety of Hsp90 clientele necessitates a diverse array of co-chaperones that help deliver clients to Hsp90 and so regulate their activation and maturation. Recent, evidence suggests that Hsp90 undergoes N-terminal dimerization involving a co-operatively driven mechanism of structural change. In the light of this, the role played by a variety of Hsp90 co-chaperones can now be reinterpreted and a better understanding of their effect on the Hsp90 cycle is beginning to emerge.

## REGULATION OF Hsp90 ACTIVITY THROUGH POST-TRANSLATIONAL MODIFICATION OF CO-CHAPERONES

Post-translational modification of co-chaperones adds further mechanisms by which Hsp90 activity can be regulated. Phosphorylation of Hsp90 co-chaperones has not been studied extensively, but its importance is demonstrated by a number of examples. Ser^13^ of human Cdc37^p50^ is the target for phosphorylation by protein kinase CK2, which appears to be necessary for kinase client chaperoning, including Cdc28^Cdc2^, Ste11^RAF^, Kin28, Mps and CK2 itself [186–188]. The phosphorylation appears to favour the formation of a Cdc37^p50^–Hsp90–kinase complex and dephosphorylation by PP5/Ppt1 appears to weaken this association and might act as a signal for progression of the cycle and release of activated client kinase protein [189].

Another example involving CK2 phosphorylation is that of Sgt1 at Ser^361^, which inhibits the dimerization of the co-chaperone [190]. This in turn influences kinetochore assembly and chromosome segregation in eukaryotes during cell division [190]. Sgt1 acts as an adaptor in several other processes such as the regulation of innate immunity systems in plants and animals and in SCF E3 ubiquitin ligase-directed protein degradation [169]. Whether this phosphorylation has a negative impact on these processes is currently unknown.

CK2 is responsible for the phosphorylation of p23 (also known as cytoplasmic prostaglandin E synthase 3) on Ser^113^ and Ser^118^, and promotes the synthesis of prostaglandin E_2_ [191]. p23 is involved in a variety of other client protein complexes including telomerase and steroid hormone receptors [192,193]. The details of how it affects their activation are currently unknown. Ser^113^ and Ser^118^ are not conserved in Sba1, the yeast orthologue of p23. In yeast, the equivalent residues are upstream of Trp^124^, which interacts with the long helix of the middle domain of Hsp90 and is thought to modulate the catalytic loop of this domain. Whether phosphorylation of human Ser^113^ and Ser^118^ affects the ability of p23 to modulate the catalytic loop of Hsp90 is unknown.

Yet another CK2-mediated phosphorylation has been seen in murine mSti1^HOP^ at Thr^189^. In contrast, Cdc2 phosphorylates mSti1^HOP^ at Thr^198^. It has therefore been suggested that mSti1^HOP^ plays a role in the cell cycle [194]. Finally, CK2 directed phosphorylation of FKBP52 is seen at Thr^143^ and is thought to play a role in steroid hormone activation [186,195]. FKBP phosphorylation at an unspecified site influences the efficiency of adeno-associated virus type-2 transduction [196–198].

For kinases, the progression of the chaperone cycle requires both Cdc37^p50^ and Aha1. The phosphorylation of Cdc37^p50^ at Tyr^4^ and Tyr^298^ was reported to disrupt client–Cdc37^p50^ association and provided directionality to the cycle [118]. In contrast, phosphorylation of Hsp90 at Tyr^197^ by the Yes kinase, was reported to cause dissociation of Cdc37^p50^ from Hsp90 [118], whereas phosphorylation on Tyr^313^ promotes recruitment of Aha1, both of which further the chaperoning process by stimulating Hsp90 ATPase activity. The phosphorylation of human Aha1 at Tyr^223^ by c-Abl kinase has been reported [199] and appears to promote its interaction with Hsp90. The increased binding of Aha1 is thought to translate into an enhanced activation of Hsp90 ATPase activity, which in turn promotes Hsp90 interaction with kinase clients. In contrast, glucocorticoid receptor and CFTR (cystic fibrosis transmembrane receptor) interactions with Hsp90 were compromised. Unexpectedly, it was reported by the same authors that Tyr^223^ phosphorylation led to ubiquitination and proteasome degradation of Aha1. Finally, Hsp90α phosphorylation at Tyr^627^ induces dissociation of the client and remaining co-chaperones that signals completion of the chaperone cycle.

In conclusion, a variety of co-chaperones are required to deliver client protein and to promote the chaperone cycle of Hsp90. The complex nature of the Hsp90 chaperone cycle has allowed co-chaperone regulation of Hsp90 by a variety of mechanisms that involve modulating the co-operative nature of N-terminal dimerization within Hsp90. Ultimately the precise regulatory effect of such co-chaperones is dependent on their post-translational modification and that of Hsp90 itself.

## CONCLUDING REMARKS

The cytoplasmic Hsp90 proteins are required for a whole host of biological processes, including adaptation to stress. It is therefore not surprising that the Hsp90 levels in cells is abundant and increases further during stress adaptation. Because of the multitude of tasks carried out by Hsp90, numerous regulatory systems operate to ensure the proper integration and regulation of Hsp90 activity. Most importantly, HSF1 emerges as a master regulator of the HSR, helping integrate a variety of cellular signals into Hsp90 transcriptional control. As such, Hsp90 is a highly regulated protein and is subject to many post-translational modifications as well as being able to sense heat stress directly. The regulation of Hsp90 is controlled by post-translational modifications, and by co-chaperones and client proteins, of which the latter are subject to various regulatory processes themselves. Although much progress has been made in understanding these processes, there remains a significant amount we still do not fully understand. For example, we have recently established some of the post-translational modifications that operate to regulate both Hsp90 and its co-chaperones; however, our knowledge of the processes that control these modifications are still in their infancy. Many of the enzymes, such as kinases, phosphatases, histone acetylases and histone deacetylases remain to be identified. Determining how these modifications are integrated into coherent regulatory systems will not be easy, but is essential if we are to understand the Hsp90 chaperone cycle in the context of the various biological processes that depend on it.

## Abbreviations

AhR: aryl hydrocarbon receptor
AMP-PNP: adenosine 5′-[β,γ-imido]triphosphate
Cdk4: cyclin-dependent kinase 4
CHIP: C-terminus of the Hsc (heat-shock cognate) 70-interacting protein
CS: CHORD and Sgt1
CTA: C-terminal transactivation
DBD: DNA-binding domain
eNOS: endothelial nitric oxide synthase
FKBP: FK506-binding protein
HDAC: histone deacetylase
HOP: Hsp70/Hsp90-organizing protein
HSE: heat-shock element
HSF1: heat-shock factor 1
Hsp: heat-shock protein
HSR: heat-shock response
IKK: inhibitor of NF-κB kinase
IL: interleukin
NF-IL6: nuclear factor for IL-6
NF-κB: nuclear factor κB
NTA: N-terminal transactivation
PP5: protein phosphatase 5
RD: regulatory domain
SP1: specificity protein 1
STAT: signal transducer and activator of transcription
Strap: stress-responsive activator of p300
TPR: tetratricopeptide repeat
UPE: upstream promoter element
